# Detecting flood-type-specific flood-rich and flood-poor periods in peaks-over-threshold series with application to Bavaria (Germany)

**DOI:** 10.1007/s00477-022-02350-8

**Published:** 2022-12-01

**Authors:** S. Fischer, D. Lun, A. H. Schumann, G. Blöschl

**Affiliations:** 1 SPATE Research Unit, Ruhr-University Bochum, Bochum, Germany; 2 Institute of Hydraulic Engineering and Water Resources Management, Vienna University of Technology, Vienna, Austria

**Keywords:** Flood-rich periods, Flood-poor periods, Scan statistics, Flood types, Peak-over-threshold

## Abstract

Previous studies suggest that flood-rich and flood-poor periods are present in many flood peak discharge series around the globe. Understanding the occurrence of these periods and their driving mechanisms is important for reliably estimating future flood probabilities. We propose a method for detecting flood-rich and flood-poor periods in peak-over-threshold series based on scan-statistics and combine it with a flood typology in order to attribute the periods to their flood-generating mechanisms. The method is applied to 164 observed flood series in southern Germany from 1930 to 2018. The results reveal significant flood-rich periods of heavy-rainfall floods, especially in the Danube river basin in the most recent decades. These are consistent with trend analyses from the literature. Additionally, significant flood-poor periods of snowmelt-floods in the immediate past were detected, especially for low-elevation catchments in the alpine foreland and the uplands. The occurrence of flood-rich and flood-poor periods is interpreted in terms of increases in the frequency of heavy rainfall in the alpine foreland and decreases of both soil moisture and snow cover in the midlands.

## Introduction

Hydrological time series such as discharge, flood or precipitation series are affected by climate and anthropogenic activities (e.g. Seibert and McDonnell 2010; Zhang et al. 2016; Prosdocimi et al. 2015; Blöschl et al. 2019b). Statistical models of time series may account for non-stationarities induced by these drivers either by adjusting the observed time series (e.g. de-trending) or by including non-stationarity in the model. In both cases, it is important to understand whether and when any non-stationarities occur and their magnitude.

Many studies in hydrology have dealt with the non-stationarity problem through trend detection and change-point estimation, using statistical tests such as the Mann–Kendall- or the Wilcoxon-test (e.g. Kundzewicz et al. 2005; Mangini et al. 2018; Garcia-Marin et al. 2020). However, results are heavily affected by the length of the observation period and the assumptions made in the analysis (Yang et al. 2005; Kundzewicz et al. 2005). One such assumption is the absence of cyclic or periodical behaviour of the stochastic processes (Koutsoyiannis 2003). Cyclic behaviour can be identified as decreasing or increasing trends if only relatively short sequences of transitions from high to low values (or vice versa) are analysed (Cohn and Lins 2005). An example of such a cyclic behaviour are flood-rich and flood-poor periods, which are usually defined as periods with an unusually large (or low) number of flood events. Typical methods (Liu and Zhang 2017; Merz et al. 2016) compare the number of observed events within a certain time span with the expected occurrence of events under the assumption of independent, identically distributed [iid] data or some other time-homogeneous stochastic process. In contrast to trends, flood-rich and flood-poor periods do not necessarily show a monotonic increase or decrease, and in contrast to change-points, e.g. in mean or variance, may vary between periods. Identifying and explaining flood-rich periods is challenging and has been named as one of the Unsolved Problems in Hydrology (Blöschl et al. 2019a).

Flood-rich and flood-poor periods can be investigated for annual maximum peak discharges [AMS] and Peak-over-Threshold series [POT]. In many flood time series, accumulations of floods or extended periods of absence of large floods are visible. This questions the assumption of iid data. However, so far, to the best of our knowledge, no consistent detection methods for these two perspectives are available in hydrology, i.e. having the same methodological approach for the different kinds of flood samples. Such tests are able to distinguish between a random accumulation and a systematic deviation from the iid assumption.

In POT-series, a flood event is interpreted as an observed (usually daily) discharge above a prescribed magnitude (implying that the prevalence of flood-rich and flood-poor periods may differ between magnitudes). The usual reference condition is a time-homogeneous Poisson Process as the null hypothesis (e.g. Mudelsee et al. 2004; Silva et al. 2012; Merz et al. 2016; Liu and Zhang 2017; Albrecher et al. 2019). Clustering beyond what could reasonably be expected from a time-homogeneous Poisson process is usually interpreted as flood-rich or flood-poor periods. The two most common methodologies for detecting flood-rich and flood-poor periods in POT series include the dispersion index (e.g. Vitolo et al. 2009) of annual flood occurrences, which can indicate clustering at the annual scale, but does not provide an estimate of the time of occurrence of the anomaly (Liu and Zhang 2017). The other common methodology proposed by Mudelsee et al. (2004) based on the non-parametric estimation of a time-varying intensity of a Poisson Process has been used in a number of studies on the clustering of floods (Silva et al. 2012; Merz et al. 2016; Liu and Zhang 2017; Albrecher et al. 2019). Merz et al. (2016) provide procedures for the evaluation of the statistical significance of this approach. The methodology requires the specification of a number of parameters including a kernel function and a bandwidth, and (optionally) the generation of pseudo-data to reduce boundary effects. Confidence intervals are built via bootstrapping and the bandwidth corresponds to the time window which is investigated with respect to clustering, but the association is not exact. Other methodologies for detecting temporal flood or more generally hydrological clustering in POT-series include regression based approaches (e.g. Villarini et al. 2013), other indices of dispersion (e.g. Serinaldi and Kilsby 2013) and approaches based on dependence of the parent process (Iliopoulou and Koutsoyiannis 2019).

In the context of AMS, Lun et al. (2020) identify flood peaks above prescribed magnitudes and suggest a procedure based on scan statistics to detect flood-rich and flood-poor periods. They use a time-homogeneous Bernoulli Process as the reference condition, because quantile exceedances of an iid-process result in a stationary Bernoulli Process (independent from the marginal distribution). A flood-rich (flood-poor) period is identified as a coherent time segment with unusually many (few) threshold-exceedances. The only parameter in this procedure is the time window. In this paper, we propose an approach to detect flood-rich and flood-poor periods based on scan statistics similar to Lun et al. (2020), but for POT-series. The AMS approach does not allow for multiple flood events within single years and thus contains less information. The approach proposed here is consistent with the one for AMS and allows the evaluation of the statistical significance of anomalies in the context of a hypothesis test with the same hypothesis as of Lun et al. (2020). Also, the time frame for the investigation of clustering can be chosen explicitly and the only input parameter is the time frame for the investigation of clustering. Here, we interpret clustering as significant deviations from a time-homogeneous Poisson process.

Additionally, we attribute the flood-rich and flood-poor periods to their generating processes by the use of flood types. There exist numerous possibilities to define flood types, e.g., focusing on the meteorological processes or catchment dynamics (Tarasova et al. 2019). Here, we apply a flood typology based on the hydrographs of the events to characterize the meteorological reasons for flood generation (Fischer et al. 2019). This flood classification is particularly useful for long time series with sparse meteorological and catchment data. However, the use of flood-type-specific time series requires the extension of the AMS theory introduced by Lun et al. (2020) as floods of a given type may not occur in each year and floods of different types may have vastly different return periods for similar discharges. Each time series of flood events of a given flood type has to be considered as POT-series with a varying number of events per year and quantile-based thresholds are applied to isolate flood events with large peaks.

The combination of statistical tests that detect flood-rich and -poor periods with flood types offers the possibility to obtain flood-type-specific information of changes, either in frequency or in magnitude. We present a methodology for detecting flood-rich and flood-poor periods, which has been extended to case of POT-series relevant for hydrological applications. The flood-rich and flood-poor periods are investigated for different flood types. The methodology is applied to 164 catchments in Bavaria, Southern Germany. The following research questions are addressed:

- Do flood-rich and flood-poor periods exist for floods of different types in Southern Germany and do they differ?
- Which were the dominating flood types of the flood-rich and flood-poor periods?
- Are small and large floods different in terms of their flood-rich and flood-poor periods and their types?
- Are the overall tendencies (increasing/decreasing frequency of small/large floods) of the detected periods consistent with previously detected flood trends from literature?

We find significant differences in the occurrence of flood-rich and flood-poor periods between flood types and how these depend on the geomorphology and hydrology of the catchments.

## Data

**Fig. 1:**
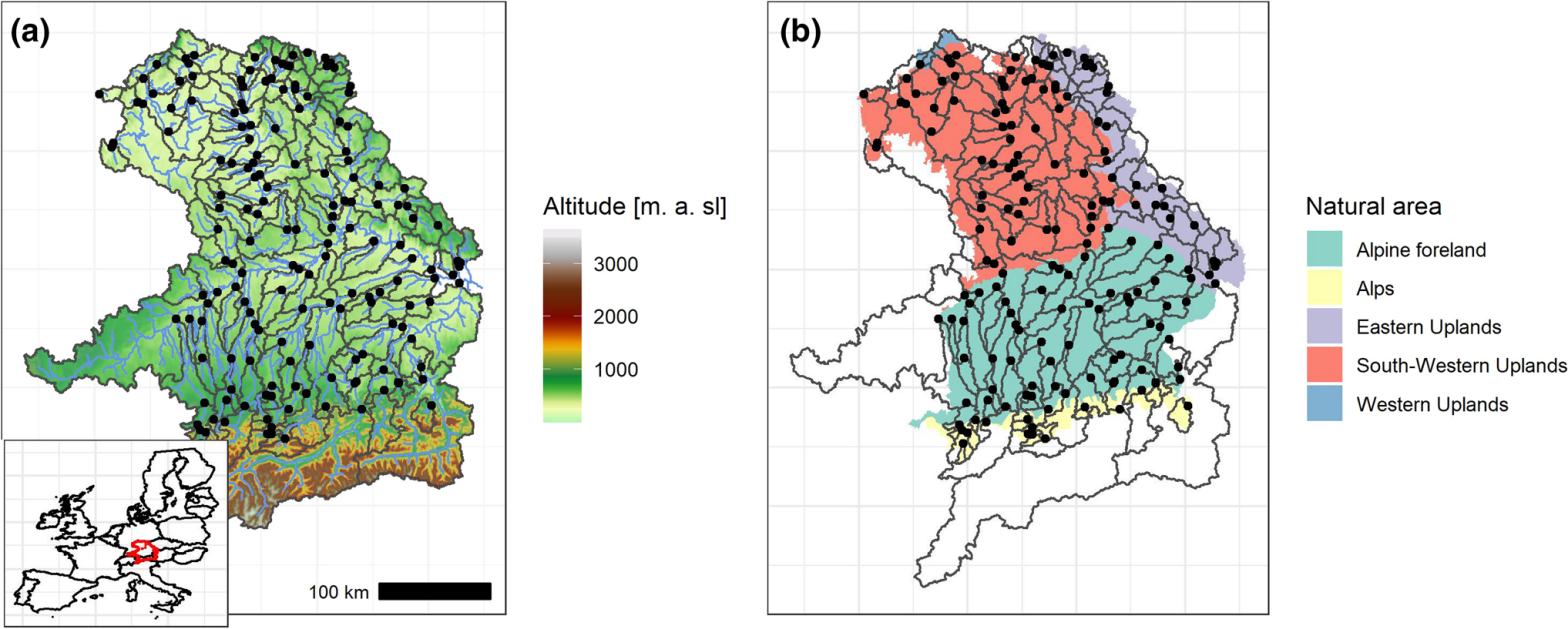
Study catchments in southern Germany with DEM (**a**) and natural area (**b**). Catchment boundaries are given in black, gauge locations are given as black dots. The inset map shows the location of the study area in Europe. For areas outside of the federal state of Bavaria, no natural areas are available and these are left blank

We considered stream gauges in Bavaria, Southern Germany. Some of the catchments extend to the Czech Republic and to Austria. Daily mean discharges as well as monthly maximum peaks (quasi-instantaneous discharges) were used in the investigation. For a catchment to be selected from the available data base, the observation period had to span at least 30 years, which applied to 164 catchments. The observation periods of the selected catchments vary between 31 and 90 years. All catchments include the period 1989–2019 and at least span the years 1930–2010. A minimum record length of 30 years was considered as this is regarded as the climate-scale, which apparently is sufficiently long for investigating clustering or trends (Dimitriadis and Koutsoyiannis 2015). Catchment sizes range from 42 to 47,518 km^2^. The physiography ranges from alpine catchments with a mean elevation of roughly 2000 m a.s.l. in the South to upland catchments in the North and East and lowland catchments with mean elevations of 300 m a.s.l. in the centre of the study region (Fig. 1a). All catchments belong to one of three major river basins: the Danube, the Main (tributary of the Rhine River) and the Saale (tributary of the Elbe River). The Federal State of Bavaria has identified five natural areas in this region (https://www.lfu.bayern.de/natur/naturraeume/index.htm), which are defined according to similarity in geomorphology, climate, hydrology, land use, flora and fauna (Fig. 1b). The natural area assigned to a catchment was defined as the natural area with the largest proportion in the catchment area. Since the “Western Uplands” only contain three catchments, which was considered to be insufficient for statistical analyses, these three catchments were reassigned to the neighbouring “South-Western Uplands” class. The catchments are distributed relatively uniformly across the natural areas, with 25 catchments belonging to the Alps, 50 catchments to the Alpine foreland, 34 catchments to the Eastern Uplands and 55 catchments to the South-Western Uplands. All catchments were checked for inconsistencies, such as the impacts of dams, by visual inspection of the discharge time series. Daily precipitation and temperature series were derived from the E-OBS 0.1-degree grid data set (Cornes et al. 2018) and, together with the elevation zones in 100 m steps, served as input for an HBV-model (Bergström 1995) to simulate daily snowmelt. We applied a lumped version of the model, where snowmelt was estimated by the degree-day method. The first year of daily discharges of each catchment was used as warm-up period, with the first 60% of the time series serving as calibration period and the last 40% serving as a validation period. For parameter optimisation, the BOBYQA algorithm (Powell 2009) was applied using 15 runs with 1000 repetitions each per catchment. An average a Nash–Sutcliffe Efficiency of 0.738 indicated a sufficient performance of the model in the validation period.

## Methodology

### Flood event separation and flood-type classification

For constructing POT series, we applied the semi-automated flood event separation algorithm of Fischer et al. (2021), which uses the 3-day-window variance of the daily discharges to define flood events. For each identified flood event, we identified the instantaneous peak from monthly maximum discharges instead of daily series to avoid smoothing effects (Ding et al. 2015). On average, between two and four events per year were identified for each catchment.

In a second step, the flood events were classified into flood types according to their hydrograph shape and the amount of snowmelt contributing to the direct runoff volume. We applied the classification of Fischer et al. (2019), which requires daily discharges, precipitation and snowmelt as input. It uses the flood timescale (Gaál et al. 2012) to distinguish between the hydrograph shapes of rainfall-induced floods (< 20% snowmelt in the total amount of runoff-generating precipitation) and applies a clustering approach to define snowmelt-impacted floods (> 20% snowmelt). For this purpose, the rainfall-induced floods are ordered according to their flood timescales. Then, the ordered sample is divided into three groups and the sum of the coefficients of determiniation of the linear regression between flood peak and volume for each group is calculated. The grouping that results in the maximum sum is then selected for the rainfall-induced flood types, where the events in the group with smallest flood timescales are given type R1 and those in the group with the largest flood timescales are given type R3. For the snowmelt-induced floods, a kMeans clustering on the sum of snowmelt, the sum of precipitation and the runoff coefficient is applied to distinguish between rain-on-snow and snowmelt-driven floods. The flood classification resulted in the following five flood types associated with different peak-volume-relationships and differing amounts of contributing snowmelt to distinguish flood-generating meteorological conditions (Fischer et al. 2019):

- R1: flood events with small volumes (i.e. flashy hydrographs), frequently associated with heavy-rainfall events of high intensity and short duration
- R2: flood events with balanced peak-volume relationships, frequently associated with medium duration rainfall of 5–10 days with medium intensity
- R3: flood events with large volume but comparably small, often multiple peaks, frequently associated with long-duration rainfall of low intensity and frequently wet soils
- S1: Rain-on-snow floods, where high amounts of rainfall occur together with snowmelt
- S2: Snowmelt-induced floods with only small contributions of rainfall.

There exist various approaches to define flood types, focusing on meteorological, atmospheric or catchment processes (Tarasova et al. 2019). Here, we chose the hybrid-hydrograph-based classification of Fischer et al. (2019). It is easily applicable to long records of discharge data, requiring only discharge, precipitation and temperature or snowmelt data for the separation of flood events. However, it does not explicitly take into account the catchment state, i.e. the soil moisture.

An overview of the (empirical) mean occurrence frequency of each flood type and the average return period is given in Table 1.

**Table 1:** Average number of flood events per year and quartiles of the return period (calculated with AMS) per flood type

Flood type	Average no. events per year	5%-Quantile return period	Median return period	95%-Quantile return period
R1	1.26	1.06	1.31	8.24
R2	0.6	1.05	1.29	7.28
R3	0.23	1.10	1.46	8.12
S1	0.57	1.06	1.39	6.51
S2	0.23	1.44	1.47	4.21

### Flood frequency analysis of flood types

Flood frequency analysis for the respective flood types, which is required for the definition of T-year thresholds for the detection of flood-rich and flood-poor periods, was performed using the method of Fischer (2018): consider a sample of flood events $X_{1}^{(j)} , \ldots ,X_{{n_{j} }}^{(j)}$ of flood type $j$, $j = 1, \ldots ,5$, with sample size $n_{j}$ for a given catchment. We define the type-specific threshold $u_{j}$ for the POT-approach as three times the weighted mean monthly discharge, where the weights are chosen according to the relative frequency of flood type $j$ in the respective month. This threshold selection was validated in previous studies to result in samples with behavior in line with extreme value theory (Fischer et al. 2019; Fischer and Schumann 2021). Of course other choices for the threshold are possible (Lang et al. 1999), e.g. according to a pre-defined mean number of events per year, a quantile or residual-life plots, though the chosen one is based on German guidelines on how to define a flood (DWA 2012). The distribution of the exceedances of the threshold $u_{j}$ of POT-series of flood type $j$, $G_{j}$, is modelled by the Generalized Pareto Distribution with parameter set $\theta_{j}$. The annual distribution function of each flood type then is derived by the total probability theorem (Cunnane 1973; Stedinger et al. 1993) as:

$$ F_{j} (x) = \sum\limits_{k \ge 0}^{{}} {{\mathbb{P}}_{j} } (l = k)(G_{j} (x,\theta_{j} ,u_{j} ))^{k} , $$

where ${\mathbb{P}}_{j} (l = k)$ is the probability that the annual number *l* of flood peaks of type $j$ above the threshold $u_{j}$ is equal to k. ${\mathbb{P}}_{j} (l = k)$ is modelled by the Poisson distribution.

### Scan statistics for peak-over-threshold series

Flood events are interpreted as realisations of a Poisson Process. A priori, we assume that there is no clustering among flood events, which we interpret as a time-homogeneous Poisson process. Flood-rich and -poor periods are interpreted as coherent periods in time for an observed POT-series with unusually many (few) flood events (i.e. clustering). How many (few) events would be statistically significance is evaluated with a scan statistic, following Glaz et al. (2001), which is equivalent to a statistical hypothesis test with null hypothesis “Homogenous Poisson process with constant intensity”.

Let $$Y(t)$$ refer to a Poisson Process with constant intensity $$\lambda$$ over the interval $$[0,T)$$, where 0 refers to the beginning of the observation period of a flood series and T to the end. For a given window $$\omega$$, let $$Y_{t} (\omega )$$ refer to the number of events in the interval $$[t,t + \omega )$$, i.e. $$Y(t + \omega ) - Y(t)$$, which follows a Poisson distribution with parameter $$\lambda \omega$$ (e.g. Theorem 6.8.2. in Grimmett and Stirzaker, 2020). Finally, let

1 $$ S_{\omega } = \mathop {\max }\limits_{0 \le t \le T - \omega } \left( {Y_{t} (\omega )} \right). $$

$$S_{\omega }$$ denotes the largest number of events observed in any subinterval of length $$\omega$$ over $$[0,T)$$ and is the continuous unconditional scan statistic. Additionally, let $$X_{(i)}$$ refer to the arrival time of the ith event and let $$W_{k}$$ be the size of the smallest subinterval containing k events

$$ W_{k} = \mathop {\min }\limits_{i \ge 1} \left( {X_{(i + k - 1)} - X_{(i)} } \right). $$

The distributions of these statistics are related

$$ {\mathbb{P}}(S_{\omega } \ge k) = {\mathbb{P}}(W_{k} \le \omega ) $$

For given $k,T,\lambda$ and $\omega$, these probabilities are denoted as ${\mathbb{P}}^{ * } (k,\lambda T,\omega /T)$. Small values of the probabilities correspond to a statistically significant result in a Likelihood Ratio test of constant intensity of events (time-homogeneous Poisson Process) versus a pulse alternative (Naus 1966; Chapter 14 and 15 in Glaz et al. 2001). The *p* value of the test is given by the probability ${\mathbb{P}}^{ * } (k_{obs} ,\lambda T,\omega /T)$, where $T,\lambda$ and $\omega$ are fixed a priori and $k_{obs}$ is obtained from an observed series of floods. It should be mentioned that the distribution of the scan statistic and the corresponding p-values is discrete; not every significance level can be obtained.

This procedure is a prospective scanning procedure and assumes that the total number of observed events in $$[0,T)$$ is itself random. For a POT-series of flood events, the total number of events is known, so for the investigation of clustering a retrospective procedure should be employed. The continuous conditional scan statistic is the same as in Eq. (1) but instead of the intensity $$\lambda$$, the total number of events N in $$[0,T)$$ is known. The corresponding probabilities are denoted as $${\mathbb{P}}(k,N,\omega /T)$$. Conditional on $$\{ Y(t) = N\}$$, the arrival times of a homogeneous Poisson Process are uniformly distributed over $$[0,T)$$ (e.g. Theorem 6.8.11. in Grimmett and Stirzaker 2020).

A period is identified as flood-rich if the corresponding p-value of the conditional scan statistic is below $\alpha = 0.05$. The magnitude of the p-values depends on the parameters of the procedure ($N,\omega$ and $T$) (T, N and k result from the data) and the choice of the window length $\omega$ is crucial. We employ a multiple window scan statistic to avoid strict assumptions on the length of flood-rich periods and account for the fact that we examine multiple window lengths at the same time (Wu et al. 2013). For window sizes $\omega_{1} < \ldots < \omega_{M}$, the distribution of the multiple window scan statistic is given by

$$ {\mathbb{P}}\left( {S_{\omega j} < k_{j} ,{\text{ for all }}j = 1, \ldots ,M} \right). $$

With $k_{1} < \ldots < k_{M}$, the complementary probability gives the p-value for the respective test for flood-rich periods. If a flood-rich period is detected via a multiple window procedure, different window sizes $\omega_{i}$ may cause this result. For the sake of presentation, the significance of these periods is then evaluated separately for the different window lengths $\omega_{i}$. Subsequently, flood-rich periods are plotted for each window length that would have led to a significant result in a single window procedure (see e.g. Fig. 3).

Flood-poor periods are defined by an improbably long absence of events, which is not symmetric to the case of flood-rich periods. By defining

$$ D_{\omega } = \mathop {\min }\limits_{0 \le t \le T - \omega } \left( {Y_{t} (\omega )} \right){\text{ and }}V_{k} = \mathop {\max }\limits_{i \ge 1} \left( {X_{(i + k - 1)} - X_{(i)} } \right) $$

and noting that ${\mathbb{P}}(D_{\omega } \le k) = {\mathbb{P}}(V_{k} \ge \omega )$ (Chapter 18.4. in Glaz et al. 2001) we can define a flood-poor period as unusually long stretches of time without events by fixing $k = 0$. No multiple windows are needed.

One major challenge in the application of scan statistics for cluster detection is their computational aspects. For given series, we look at the k + 1-th order gaps $W_{k}$ and $V_{k}$ to avoid scanning infinitely many windows. Given the longest window without events, the p-values for flood-poor periods can be calculated exactly (for the case with no exceptions, i.e. k = 0), see e.g. Parzen (1960, p. 306). For flood-rich periods, this is not the case. Whereas the distribution of the discrete scan statistic for observations of a Bernoulli Process can be evaluated exactly (Fu 2001), for the continuous scan statistic, applicable to realizations of a Poisson Process, the distribution can only be evaluated exactly for restricted ranges of parameters (Fu et al. 2012). Numerous approximations exist (Naus 1982; Chapters 10 and 11 in Glaz et al. 2001) and the distribution of the continuous scan statistic is the limiting distribution of a discrete scan statistic (Fu et al. 2012; Wu et al. 2013), but this approach is computationally expensive. Therefore, we rely on simulations to produce p-values for the detection of flood-rich periods (nsim = 10,000). The accuracy of this procedure was tested and compared with approximations (namely the approximations from Naus 1982), yielding reliable results, and the number of simulations is increased for observed p-values close to the significance level (nsim = 100,000).

**Fig. 2:**
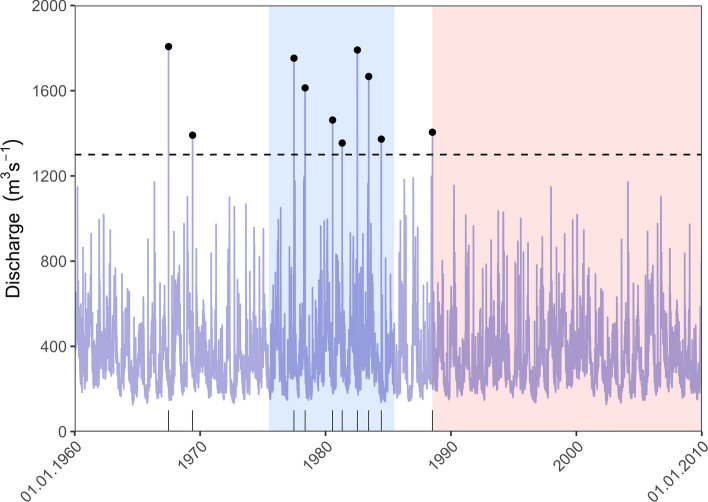
Illustration of the conditional scan statistic applied to POT-series. Series of daily discharges (blue in background) and events (here as an example: discharges over 1300 m^3^ s^−1^) plotted as black points and bars at bottom. Blue background indicates a detected flood-rich period, red background indicates detected flood-poor period

We illustrate the procedure by a simple example of N = 10 observed events over the interval $\left[ {1960,2010} \right]$, representing 51 years of data (Fig. 2). To facilitate the reproducibility of this example, the event times can be found in Table 2. We apply a threshold of 1300 m^3^/s and use a window of 10 years to scan for flood-rich periods. The largest number of events found in such a window is 7, in a period stretching from 1975 to 1985. In continuous time, we observe infinitely many such windows, only one is shown in Fig. 2, for the sake of presentation. The corresponding p-value is roughly 0.02, indicating that observing such a period is rather unlikely for a homogeneous Poisson Process. The tail probabilities of the corresponding scan statistic for different values of k in this example can be found in Table 3 in the “Appendix”. We observe a period of roughly 21.5 years without events at the end of the series. The probability of observing a flood-poor period of at least this length (*p* value) in a realization of a homogeneous Poisson Process is roughly 0.04.

## Results

The method of scan-statistics for POT series was applied to each flood type and catchment separately. Each flood-type-sample was treated as a POT series. This way, type-specific results were obtained. The window lengths for the detection of flood-rich periods were chosen as 10, 20 and 30 years to detect the different spectra of flood-rich periods. Since the minimum length of the observation period of all catchments was 31 years, longer windows were not considered for comparability. We considered a significance level of 5%, which is statistical standard.

**Fig. 3:**
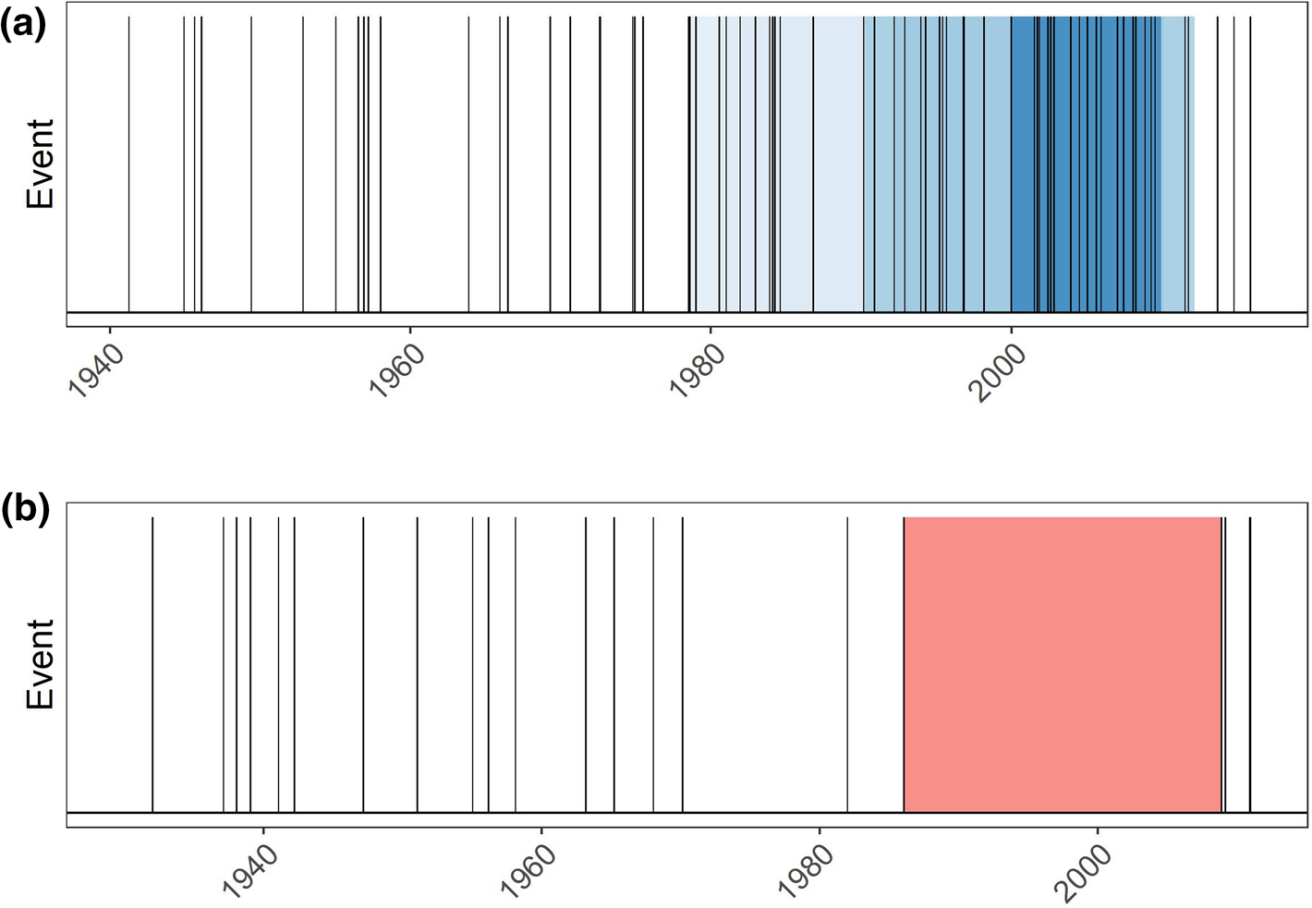
Examples of detected flood-rich and -poor periods (5% significance level) for flood type R1 (floods caused by heavy rainfall) at Unterköblitz/Naab gauge (**a**) and for flood type S2 (snowmelt floods) at Leucherhof/Baunach gauge (**b**). Vertical lines represent occurrence of flood events of the respective flood types in the given years on the x-axis. The red-shaded parts are the detected flood-poor periods, while the blue shaded parts are the detected flood-rich periods. The different shades of blue indicate the different window length 10, 20 and 30 years (from dark to light blue)

Examples for the detection of flood-rich and -poor periods are given in Fig. 3, where two gauges were selected, Unterköblitz at the Naab River (a tributary of the Upper Danube) and Leucherhof at the Baunach River, a tributary of the upper Main. The first catchment belongs to the natural area “Alpine foreland”, while the latter belongs to the “South-Western Uplands”. In Fig. 3a, for short-rainfall floods (R1), in the years 1980–2015 there occurred a significant flood-rich period. This flood-rich period was mainly driven by the elevated frequency of events in the years 2001–2010, since for this period all three window lengths, 10, 20 and 30 years, simultaneously delivered a significant flood-rich period. The whole period between the years 1980–2015 was significant for this flood type. In Fig. 3b, the opposite is demonstrated for flood type S2, which is associated with snowmelt-impacted floods. Here, a flood-poor period was detected in the period 1985–2009.

### Temporal occurrence of flood-rich and -poor periods

**Fig. 4:**
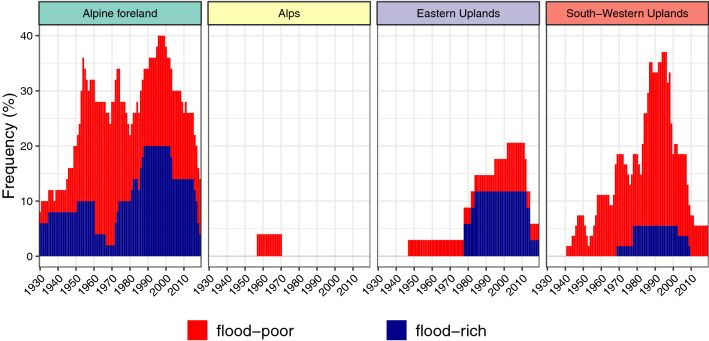
Relative frequency (number of stations with anomaly in the respective year divided by the number of all stations with data in this period) of significant flood-rich and flood-poor periods for all flood events in the study area stratified by natural areas. Flood-rich periods were identified using 10-, 20-, and 30-year-windows. To avoid multiple counting, a significant flood-rich period was counted only once, no matter how many windows were significant for the respective year. Annual frequency of catchments with flood-poor periods is stacked on top of flood-rich periods

For the first analysis, we considered all flood types jointly (Fig. 4) to facilitate the comparison with existing trend studies.

Figure 4 demonstrates that the highest number of flood-rich and flood-poor periods were identified in the Alpine foreland. In the last 40 years, flood-rich periods were detected in up to 20% of all catchments and a similar percentage of catchments showed flood-poor periods. In the years between 1930 and 1950, few flood-rich and flood-poor periods were detected in 4% and 10% of the catchments, respectively. Similarly, in the Eastern Uplands, flood-rich periods only occurred in the recent decades. In the Alps, a flood-poor period was detected for one gauge only. In the South-Western Uplands, flood-poor periods tend to dominate over flood-rich periods. The detected flood-rich and flood-poor periods are not overly sensitive with respect to the chosen window size: similar results are obtained when changing the window sizes to 7 years (and its multiples) (Fig. 8 in the “Appendix”), a window length associated with the cyclic occurrence of the El-Nino Southern Oscillation (Fredriksen 2020).

**Fig. 5:**
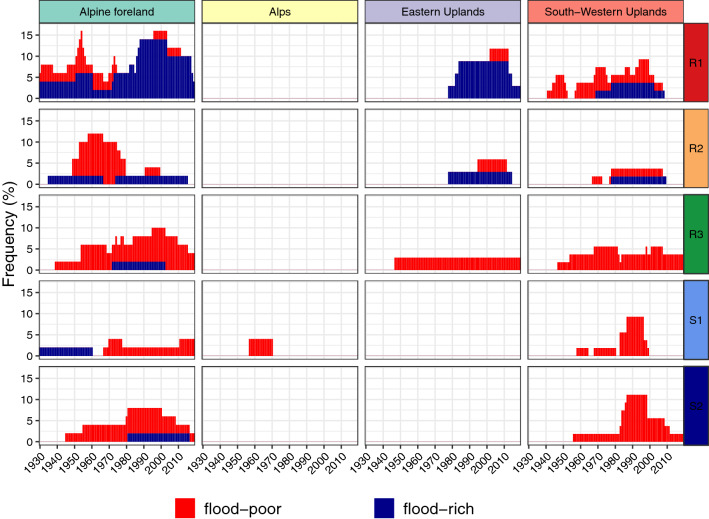
Same as Fig. 4 but stratified by flood type

In order to shed light on the generating mechanism, we stratified the floods by their flood types (Fig. 5). Overall, Fig. 5 gives a similar pattern as Fig. 4, but there are clear differences between the flood types.

For short rainfall floods (R1), which are (frequently) related to heavy-rainfall events, flood-rich periods were detected with elevated frequency for the most recent 30–40 years for catchments in the Alpine foreland (12%), Eastern Uplands (8%) and South-Western Uplands (4%). For the Alpine forelands, flood-rich periods of this flood type also occurred in the early years of the observation period, though less frequently. Flood-poor periods of R1-floods occurred rarely, some of them around 1950. A significant number of catchments with flood-poor periods of medium-duration rainfall-floods (R2), occurred between 1950 and 1980 in the Alpine foreland. For long-duration rainfall-floods (R3), mostly flood-poor periods occurred. Again, the largest number of catchments with such periods was in the Alpine foreland, where the number of anomalies increased between 1990 and 2010. For the South-Western Uplands, almost constant frequencies of about 5% occurred. Similarly, for the snow-impacted floods only flood-poor periods emerge. For rain-on-snow floods (S1), the highest frequencies of flood-poor periods occurred around 1990, mostly in the South-Western Uplands. For snowmelt-floods (S2), large numbers of flood-poor periods were detected in the period 1985–2010 for both South-Western Uplands (12%) and Alpine foreland (8%). When differentiating between the window sizes considered in this study for the detection of flood-rich periods, it becomes evident that a higher number of catchments with a flood-rich period of short-rainfall floods in the Alpine forelands had a significant period of 30 years compared to those with a 10-year-period (Fig. 9 in the “Appendix”). For this region, long-term changes appeared.

The results revealed that there exist significant differences between the flood types. While for heavy-rainfall-induced floods mostly flood-rich period occurred in the recent years, snow-impacted floods and especially snowmelt-floods were characterised by flood-poor periods for the same period.

### Spatial occurrence of flood-rich and -poor periods

**Fig. 6:**
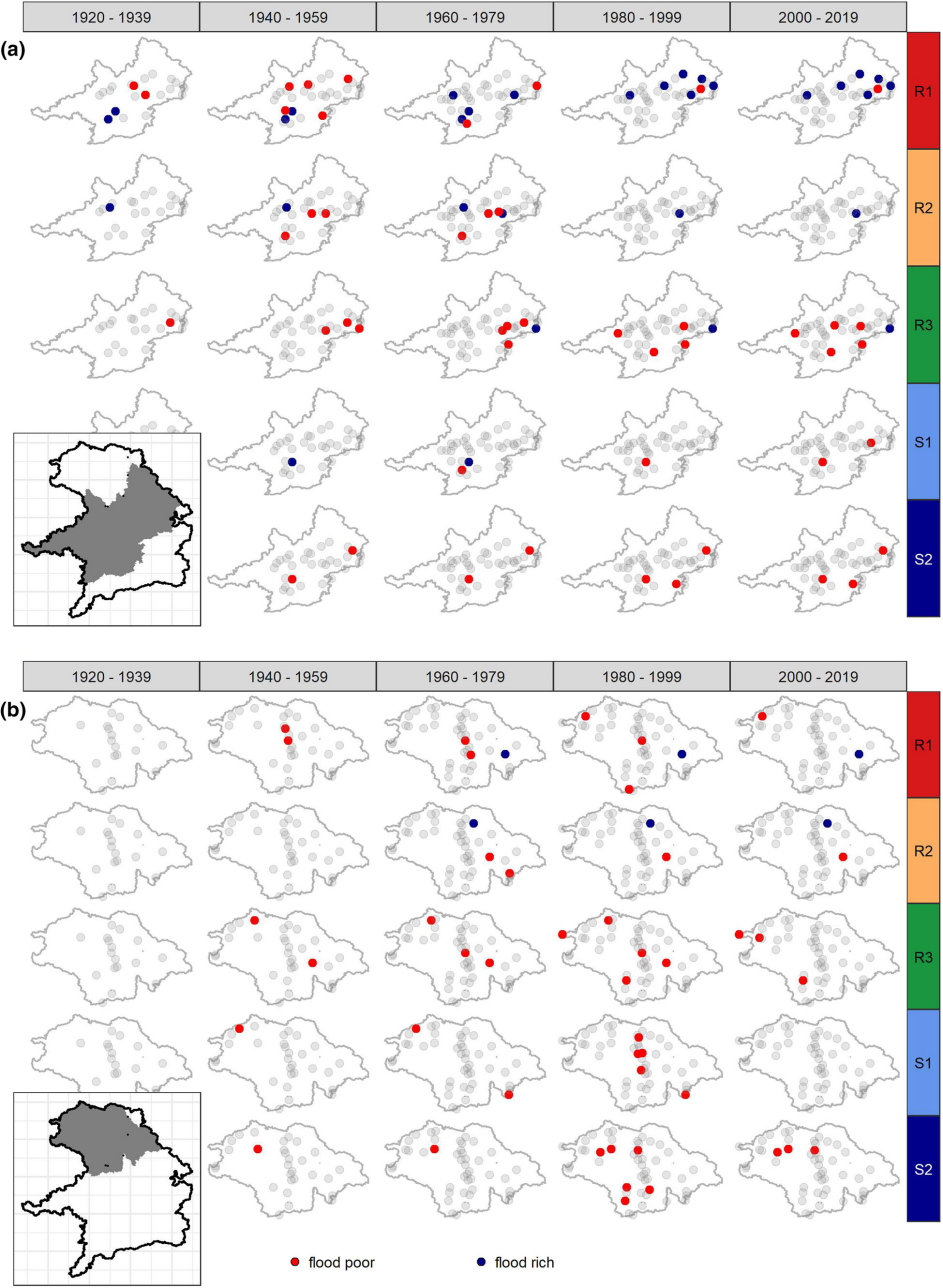
Gauges with flood-rich or flood-poor periods stratified by flood type and 20-year time periods for the Alpine foreland (**a**) and the South-Western Uplands (**b**). A catchment was labelled “flood-rich” or “flood-poor” if at least for one year in the 20-year period such an anomaly occurred. Light grey dots are gauges without such an anomaly in the respective period. Inset maps show where the catchments are located in Bavaria (in grey)

In order to analyse the spatial patterns in more detail, Fig. 6 shows maps of flood-rich and flood-poor periods for two subregions of the study area. Figure 6a reveals that the flood-poor periods of short rainfall-floods (R1) in the period 1930–1960 detected in Fig. 5 occurred in the central parts of the Danube basin in the Alpine foreland, while the flood-rich periods were more concentrated on catchments in the lower part of the Danube close to the German-Austrian border in the period 1980–2020. This implies that there exist spatial differences of flood anomalies within a natural area. The flood-poor periods in the period 1930–1960 of medium-duration rainfall-floods also occurred in the central parts of the Danube basin, while the flood-poor periods of long-duration rainfall floods in the period 1950–2010 occurred in the lower Danube basin. For the South-Western Uplands (Fig. 6b), the flood-poor periods for the snowmelt floods were especially pronounced. For both snowmelt flood types, these periods occurred between 1980 and 2010 in the central parts belonging to the flatter parts of the Main basin.

### Occurrence of flood-rich and -poor periods for large flood peaks

**Fig. 7:**
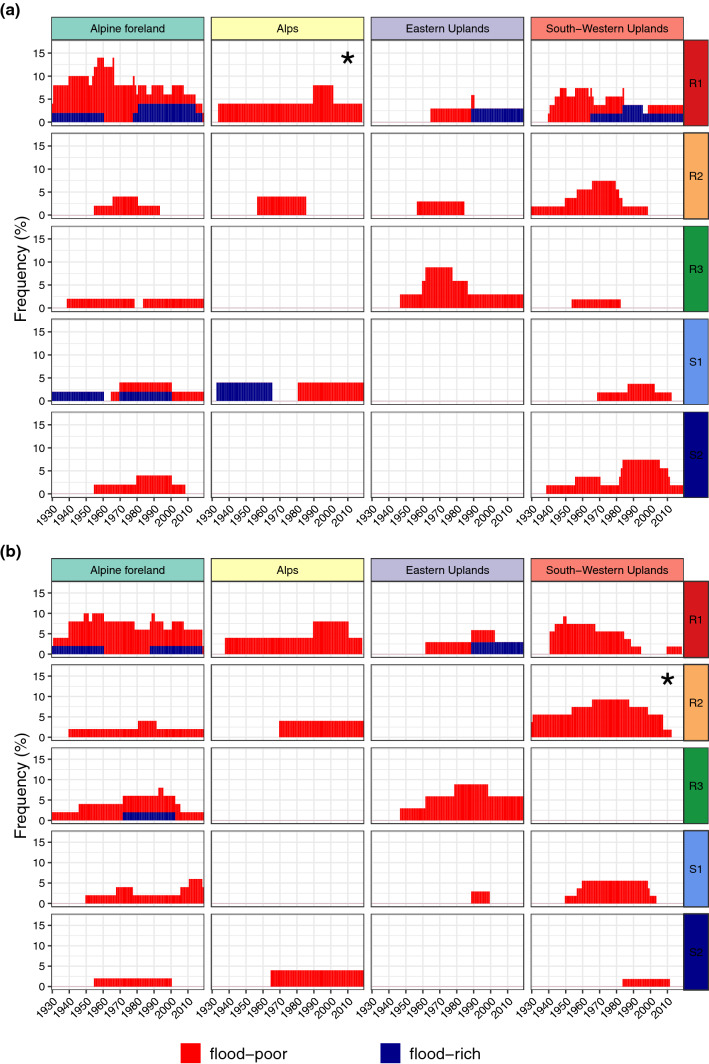
Relative frequency (number of stations with anomaly in the respective year divided by the number of stations with data in this period) of significant flood-rich and -poor periods for all flood events in the study area stratified by flood-type (rows) and natural area (columns) with application of 5-year flood thresholds (**a**) and 10-year flood thresholds (**b**). Flood-rich periods were identified using 10-, 20-, and 30-year-windows. If a flood-rich period was detected for any of these windows, the respective year was counted. Annual frequency of catchments with flood-rich is stacked on top of flood-poor periods. The asterisks highlight the most pronounced differences from the case without quantile-threshold (Fig. 5)

**Fig. 8:**
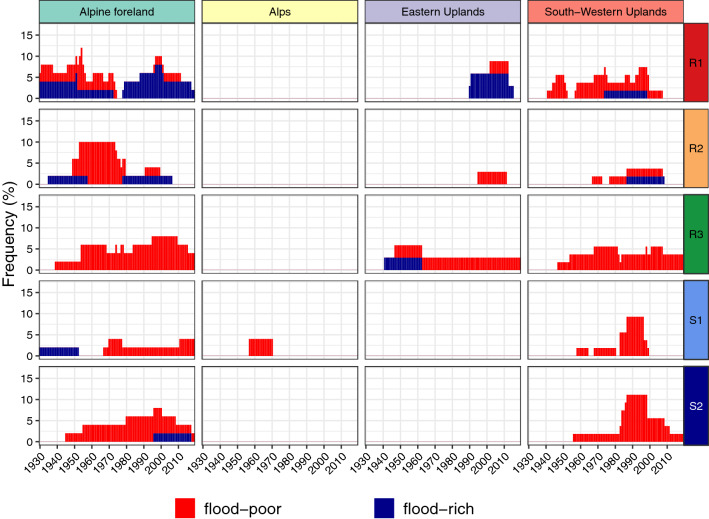
Same as Fig. 5 but with window lengths 7, 14 and 21 years

**Fig. 9:**
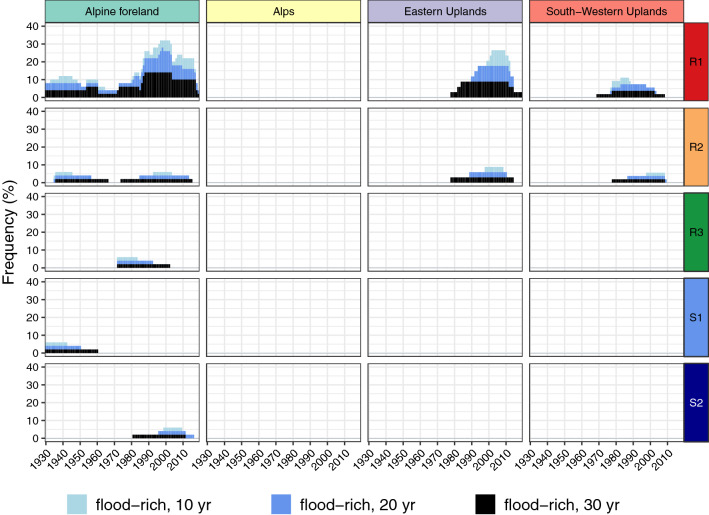
Same as Fig. 5 but with separate display of the significant 10-, 20-, and 30-year flood-rich periods. Flood-poor periods are omitted due to readability

So far, we investigated flood clustering for all available flood events, defined by threshold-exceedances of daily discharge series. In this section, we apply higher thresholds to the samples and only consider flood peaks above these thresholds. We use two thresholds corresponding to floods with 5- and 10-year return period of the respective flood type. More specifically, we used the statistics given in Sect. 3.2 to estimate the type-specific quantile corresponding to a return period of 5 respectively 10 years. All events with flood peaks above this threshold were analysed for flood-rich and -poor periods (Fig. 7). This application is similar to the one proposed in Lun et al. (2020), where T-year thresholds were used to investigate flood-rich and -poor periods in the AMS. In contrast to our previous results, here the focus is on large events.

In general, the number of catchments affected by flood-rich periods decreases with increasing threshold. These findings were already reported by Lun et al. (2020), who explained them by the higher sensitivity of the Scan-statistics to the thresholds and the decreasing availability of data for the detection of clustering. The general tendency is maintained for all thresholds: For short-rain floods (R1), flood-rich periods were mostly detected in the last 30–40 years, while flood-poor periods occurred with higher frequency in the period 1940–1970. The number of catchments affected by flood-poor periods of snowmelt floods (S2) decreases with increasing thresholds, implying that large snowmelt-floods did not show flood-poor periods as often as small floods. However, snowmelt-floods rarely produce large peaks in general (Table 1).

## Discussion

### Does a coherence between trends and flood-rich or -poor periods exist?

Several studies analysed trends in the study region. Petrow and Merz (2009) investigated AMS and POT series (3 events per year) of either annual, winter or summer flood peaks in Bavaria in the period 1951–2002. Applying the Mann–Kendall test with a 10%-significance level, the authors found increasing trends for several catchments in the Danube (POT, AMS) and Main (AMS) river basins, in both winter (Danube, Main) and summer (Danube). This is consistent with our findings of flood-rich periods of short-rainfall floods (R1), mainly in summer, for the Alpine foreland and the Eastern Uplands in the last 30 years, as these flood-rich periods at the end of the observation period can be interpreted as trends. Mangini et al. (2018), who considered AMS and POT (1–6 six events per year) flood series in the period 1965–2005, similarly reported increasing trends (10% significance level) in small alpine catchments in southern Bavaria (AMS) and generally increasing trends for the magnitude but not the frequency (POT) of flood peaks in Bavaria. Again, their results are consistent with ours, since we found several flood-rich periods in the Alpine foreland, the Eastern Uplands and the South-Western Uplands at the end of the observation period (1985–2018), which can be interpreted as trends. Hattermann et al. (2019), who considered AMS flood series in the period of 1951–2008 using the Mann–Kendall test (10% significance level), also found increasing trends for catchments in the lower Danube and the Main river basin in Bavaria, but also decreasing trends for catchments in close proximity to the higher regions of the Alps. These decreasing trends can be linked to the flood-poor periods we found for the snow-impacted flood events in the Alpine foreland in the period 1980–2020. A reason, why these trends have been evaluated as significant by Hattermann et al. (2019) but not in the previously mentioned studies may be the longer observation period of Hattermann et al. (2019). Our results also imply a change in the timing of floods especially for the Alps and alpine foreland. While snow-melt floods in winter and spring now occur less often (increasing frequency of flood-poor periods for floods of type S1 and S2, see Fig. 5), the frequency of short-rainfall floods in the summer has increased (increasing frequency of flood-rich periods for floods of type R1, see Fig. 5). This shift in the timing of floods from winter to summer in the Alps and the alpine foreland is in line with the seasonality analysis of Blöschl et al. (2017) based on annual floods. The detected flood-rich and flood-poor periods are also consistent with the findings of Lun et al. (2020) for AMS series in Southern Germany. The detected flood-rich periods for large flood peaks are consistent with our findings of flood-rich periods of heavy-rainfall floods, which are often associated with high flood peaks (Fischer et al. 2019).

### Which were the mechanisms causing the flood-rich and flood-poor periods?

When linking the detection of significant flood-rich and -poor periods to the five flood types, interesting patterns appeared that can shed light on the underlying flood-generating processes. The flood types and their hydrological interpretation are discussed in detail in Fischer et al. (2019). In our manuscript, we present an analysis related to this topic, as we investigate flood clustering for floods of these flood types. Our results indicate that flood clustering manifests very differently for different flood types (e.g. Fig. 5). For example, flood-rich periods occurred for many catchments in the Alpine foreland in the most recent decades for short-rainfall floods (R1) (Fig. 5). These results are in line with analyses of Winterrath et al. (2017), who found an increasing number of heavy rainfall events—as defined by the German Weather Service (DWD)—in this region. They suggest that this increase has a direct impact on the flood risk in this region. The detected periods are comparably long with a period of 30 years, which is similar to many studies on cyclic behaviour of extreme rainfall around the globe (see Gregersen et al., 2015, and the references therein). This granularity of our results would not be achievable, if we did not account for different flood types in our POT-series (like in e.g. Merz et al., 2016 or Liu and Zhang, 2017) or if we worked with different data, e.g. annual maxima (Lun et al., 2020).

Catchments affected by flood-rich periods of short-rainfall floods (R1) are mainly concentrated in the Danube basin at the northern fringe of the Alps, where Vb storm tracks (van Bebber 1882) are often relevant for major floods (Hofstätter et al. 2016, 2018). Vb storm tracks carry atmospheric moisture from the Mediterranean to South-East Germany and the surrounding regions (Blöschl et al. 2013). Hofstätter and Blöschl (2019) have found that cyclones associated with Vb tracks tend to cluster in time.

For medium-duration rainfall floods (R2), no pronounced spatial patterns of flood-rich and -poor periods were detected. However, several significant flood-poor periods occurred in the most recent years in the South-Western Uplands for floods with large peaks. This may be related to drier soils resulting from higher evaporation (Quesada et al. 2012; Copernicus 2018).

For long-duration rainfall-floods (R3), for all natural areas except the Alps, significant flood-poor periods were detected in the last decades. One explanation may again be a tendency of the region towards drier soils. Dry soils may be more relevant for medium-duration floods than for long-duration floods as there is less opportunity for the soil to wet up during the event (Grillakis et al. 2016).

Flood-poor periods occurred in the last 30–40 years for snow-impacted floods (S1 and S2), (Figs. 5, 7), especially in the flat parts of the Main river basin. These results can clearly be explained by the shallower snow packs in the low elevation catchments of this region (Kreyling and Hunry 2011), which tends to reduce snowmelt. The reduced frequency of snowmelt and rain-on-snow floods floods can have an impact on reservoir operation and water availability. Normally, spring floods with large volumes fill the reservoirs but this may now occur less frequently, thus potentially causing water shortages. However, this only has an impact on low elevation catchments. For high-elevation catchments, long-lasting snow packs still occur and hence snow-induced floods (S1 and S2) occur as frequently as at the beginning of the last century.

### Are small and large floods different in terms of their flood-rich and flood-poor periods and their types?

We compared the occurrence of flood-rich and flood-poor periods for small and large floods by considering all flood events of a flood type in one case, and only those above a threshold corresponding to either the 5-year or 10-year flood in another case. The results indicate that the occurrence of such periods indeed depends on the peak magnitude. For short-rain floods (R1), flood-rich periods were detected for all flood events of this type for almost all natural areas. However, when only considering events with peaks larger than the 10-year flood, significant flood-rich periods were no longer detected. This implies that, although there exist periods with an unusually large number of floods of a given type, this is not the case for the largest floods. This finding is important in a flood management context, as the frequent reoccurrence of large floods completely changes flood management policies (Viglione et al. 2014). For long-duration rainfall floods (R3), the detected flood-rich periods of all floods and large floods were similar, indicating that these flood-rich periods are mainly caused by large flood peaks.

Flood-poor periods occurred for both large and small flood peaks. In the Alps, flood-poor periods for the large floods tended to occur more frequently in recent years. Both short-rain floods and snowmelt-floods were involved in this change. In other natural areas, there was also an increase in flood-poor periods for large floods, especially for the years 1950–1990. During this time period, flood-poor periods of short- and medium-duration-rainfall floods (R1 and R2) were detected in the South-Western Uplands and flood-poor periods of flood type R3 (associated with long-duration rainfall) in the Eastern Uplands. However, this phenomenon does not continue into recent years, instead, the number of flood-poor periods of these flood types decreases for most natural areas.

Overall, these results emphasize the need for treating changes in the frequency and the magnitude of floods differently, in particular when considering flood types.

### Limitations of the proposed methodology

The flood events considered here have been identified and classified by the approach of Fischer et al. (2021) and Fischer et al. (2019). There are many other possible flood typologies (Tarasova et al. 2019). Limitations of the separation and classification approaches applied here are discussed in Fischer et al. (2021) and Fischer et al. (2019).

The proposed methodology based on scan-statistics has several advantages compared to existing methods: it is easily adaptable to different flood magnitudes, it provides a statistical test, it can be applied to several different window lengths simultaneously, and it can be applied to both POT and AMS. However, there are also limitations to be considered.

First, when treating all catchments jointly, the problem of multiple testing and spatial dependency arises, as in the case of trend tests. Multiple hypothesis testing may result in a large number of incorrect rejections of local null hypotheses if not accounted for. Often, this problem is avoided by applying the Bonferroni-rule, where the p-value of each test is divided by the number of all tests, or the false discovery rate (Benjamini and Hochberg 1995). However, these procedures require a continuous distribution of the local test statistics, which is not the case for the scan-statistic. Other approaches, such as the false detection rate for discrete test statistics require the entire distribution of the test statistic (Chen et al. 2018), which is computationally expensive. Similar to Lun et al. (2020), we therefore decided to apply the scan-statistics, keeping in mind that the results may be affected by multiple testing. The overall high frequency of catchments with detected flood-rich or flood-poor periods suggests that there is significance in the results despite spatial correlation.

Another consideration is the high dependence of the outcome on the observation period which is a problem with almost all clustering procedures. If the observation period is too short or the observation period begins or ends during a flood-rich or flood-poor period, the test may not be able to detect it. To reduce the associated uncertainty, we defined a minimum observation length of 30 years in this paper which is regarded as the climate-scale and thus also defines the maximum length that should be investigated for the detection of clustering (Dimitriadis and Koutsoyiannis 2015).

Finally, only one flood-rich and one flood-poor period is considered for each series. In reality, several such anomalies may occur, e.g. caused by cyclic behaviour. In its current form, the proposed test is not designed to detect multiple occurrences, but it is possible to extend the methodology to analyse an unusually high number of non-overlapping clusters with elevated event frequency (Section 17 in Glaz et al., 2001).

### Clustering and dependence

The reference condition for the detection procedure of flood-rich and flood-poor periods in this paper is a time-homogeneous Poisson process, implying a time-constant intensity of events, defined as threshold exceedances, as well as their independence in time (independence of increments of the process). This reference condition is usually motivated by asymptotic results for peak-over-threshold series derived from a stationary process, such as mean daily discharges (Coles et al. 2001), frequently used in hydrological applications (Lang et al. 1999) and has been used for detecting flood-rich and flood-poor periods in other publications (e.g. Liu and Zhang 2017; Merz et al. 2016). Here, two potential issues arise: Firstly, asymptotic results might not be adequate in hydrological applications, such as threshold exceedances derived from daily flows. Secondly, while a time-homogeneous Poisson process for the arrival times of threshold exceedances is an asymptotically valid model under some (short-range) dependence conditions of the underlying process (Novak 2019), this might no longer be the case for long-range dependent processes. A common procedure to obtain approximately independent events is declustering (p. 99 in Coles et al. 2001): Threshold exceedances that are close in time are interpreted as a cluster, and only one exceedance of the cluster is counted as an event. Here, some process-based knowledge can inform the formation of clusters (e.g. the recession time of a flood-event hydrograph, see e.g. Lang et al. 1999). However, even after applying such a filtering procedure, some dependence can remain in the resulting exceedance process, especially in the case of long-range dependence of the underlying process.

Mathematically, the clustering of threshold exceedances in the present context can be explained both via a non-stationary signal (as modelled in the procedure of this manuscript) as well as dependence among events, due to dependence in the underlying process. The latter possibility is explored in detail in Iliopoulou and Koutsoyiannis (2019), where the authors point out that the resulting clustering in POT-events can be explained by long-range dependence of the underlying process from which the threshold exceedances are derived. Long-range dependence is known to manisfest in persistent behaviour of the time series. Given that time series of many natural phenomena show behavior suggesting long-range dependence of the underlying processes (Dimitriadis et al. 2021), persistence offers an alternative explanation to non-stationarites for the clustering of extremes (if clustering is defined as patterns that are inconsistent with an iid-process). Yet, long-range dependence remains hard to detect in hydrological time series, especially when the observation period is short (Barunik and Kristoufek 2010). Whether or not long-range dependence occurs in hydrological time series, and if it is pre-asymptotic behaviour only or whether any physical processes exist that can explain such a statistical model, remains a frequently discussed topic (e.g. Klemes 1974; Salas et al. 1979; Mesa and Poveda 1993; Beran 1994; Koutsoyiannis 2011).

## Conclusions

Several previous studies have raised concerns that flood-rich and flood-poor periods may exist in discharge series. These anomalies may substantially affect the statistical and deterministic modelling and investigation of floods. In this paper, scan-statistics for POT-series are combined with flood types to detect and attribute these periods. With this approach, we propose a statistical test for the detection of flood-rich and flood-poor periods in POT-series. Additionally, we discuss possible underlying hydrological and meteorological mechanisms for the observed flood-rich and flood-poor periods. Clustering in this study corresponds to the timing of flood occurrences being inconsistent with a time-homogeneous Poisson process. Of course, other assumptions on clustering may also be valid but are not covered by this approach.

The results show evidence for the existence of flood-rich and flood-poor periods in the POT series in Southern Germany. However, the occurrence of such periods depends on the location and the flood type. The results also revealed that there is an increase in the occurrence of heavy-rainfall floods in the most recent years while snow-impacted floods and those caused by long-duration rainfall decreased in frequency. This is in line with several studies, including studies that indicate a shift in the seasonality of annual floods (Blöschl et al. 2017; Tabari 2020), and climate projections. These shifts in timing and the changes in the hydrograph shape of the floods as indicated by the flood types have a crucial impact on flood risk. In line with these results, flood frequency increases in summer for several catchments in the study area with large peaks and heavy-rainfall floods, while in spring there will be fewer floods with large volumes and therefore reservoirs may no longer be filled. Possible reasons may be an increase of the frequency of heavy-rainfall in this region (Winterrath et al. 2017) and decreasing soil moisture in lower elevation catchments (Quesada et al., 2012), though this remains speculative.

The flood anomalies detected here are consistent with trend studies in the study region. It seems that flood-rich periods at the end of an observation period and increasing trends are often correlated, so one can easily be interpreted as the other. Similarly, flood-rich periods at the beginning of an observation period correlate with a decreasing trend. It is clear that the observation period plays a crucial role as it can mask cyclic behaviour.

This research has revealed patterns of flood-rich and flood-poor periods aligned with changing hydrological and meteorological conditions in the study region. The lines of reasoning presented here primarily apply to the study area in Bavaria. The next step could be to extend the study region, e.g., to Europe, similar to Lun et al. (2020) but for POT-series and with attribution of these anomalies. Moreover, additional data such as soil moisture could be considered to explain the detected flood-poor periods in more depth.

## Appendix

See Tables 2, 3 and Figs. 8, 9.

**Table 2:** Event times of flood discharges over 1300 m^3^ s^−1^ in Fig. 2

Event	1	2	3	4	5	6	7	8	9	10
Date	1967-06-26	1969-05-26	1977-06-26	1978-05-16	1980-07-25	1981-04-30	1982-07-16	1983-06-11	1984-06-12	1988-07-10

**Table 3:** Distribution of the continuous conditional scan statistic for the example in Fig. 2 given by the tail probabilities ${\text{P}}\left( {{\text{k}},{\text{N}},{\upomega }/{\text{T}}} \right)$

*k*	1	2	3	4	5	6	7	8	9	10
$P_{Approx}$	1.0000	0.9998	0.9875	0.8137	0.4021	0.1150	0.0205	0.0023	0.0001	0.0000
$P_{Sim}$	1.0000	1.0000	1.0000	0.9030	0.4335	0.1184	0.0201	0.0022	0.0001	0.0000

Probabilities correspond to observing at least k events in a window of length ${\upomega }$, given ${\text{N}}$ events over the period $\left[ {0,{\text{T}}} \right)$. For the example: ${\text{N}} = 10$, ${\text{T}} = 18263$ (number of days from 1.1.1960 until including 31.12.2009) and ${\upomega } = 10\times 365$. ${\text{P}}_{{{\text{Approx}}}}$ corresponds to the probabilities obtained by the approximation in Naus (1982) and ${\text{P}}_{{{\text{Sim}}}}$ corresponds to probabilities obtained via simulations (n_sim_ = 100,000). All numbers are rounded to 4 digits
